# Determinants of Maximal Oxygen Consumption in Cardiac Rehabilitation Patients: The Role of Sex

**DOI:** 10.3390/jcm15020904

**Published:** 2026-01-22

**Authors:** Teresa Gisinger, Robert Berent, Eleonore Pablik, Nagihan Kilic Kanyücel, Fatih Kanyücel, Jürgen Harreiter, Alexandra Kautzky-Willer

**Affiliations:** 1 Unit of Gender Medicine, Division of Endocrinology and Metabolism, Department of Internal Medicine III, Medical University of Vienna, Waehringer Guertel 18-20, 1090 Vienna, Austria; 2 HerzReha Bad Ischl, 4820 Bad Ischl, Austria; 3 Section for Medical Statistics, Center for Medical Statistics, Informatics, and Intelligent Systems, Medical University of Vienna, 1090 Wien, Austria; 4 Department of Medicine, Landesklinikum Scheibbs, 3270 Scheibbs, Austria

**Keywords:** cardiac rehabilitation, sex differences, VO_2_ max

## Abstract

**Objectives**: We aimed to assess sex differences in benefits from cardiac rehabilitation and the impact of comorbidities. **Methods**: We analyzed 3239 individuals with cardiovascular diseases (81.2% males) who participated in a three-week cardiac rehabilitation program at Bad Schallerbach center (Upper Austria). Training success was measured by maximal oxygen consumption (VO_2_ max). Sex-specific differences in baseline characteristics were assessed using *t*-tests/chi^2^ tests. Associations between covariates and the outcome were evaluated with baseline-adjusted univariate analysis of variance/linear regression models. Covariates significant at α = 0.05 were included in a multivariable linear regression model, which was refined by backward selection based on the best Akaike information criterion. The final model was used to test the relationship between sex and the outcome. **Results**: The mean age and BMI were 63.9 years and 27.5 kg/m^2^ for males and 67.2 years and 27.4 kg/m^2^ for females. Males had higher baseline VO_2_ max compared to females (26.18 mL/min/kg vs. 23.55 mL/min/kg, *p* < 0.001), and a greater change in VO_2_ max after rehabilitation was seen in males compared to females (3.64 mL/min/kg vs. 2.77 mL/min/kg, *p* < 0.001). Female sex was associated with a 1.4-point-lower change in VO_2_ max after adjustment for comorbidities, sex, and training intensity (β coefficients = −1.409; CI 95% −0.410, −0.104; *p* < 0.001). Heart valve surgery (β coefficients = −0.90; CI 95% −1.444, −0.366; *p* < 0.001) and diabetes mellitus (β coefficients = −1.207; CI 95% −1.926, −0.488; *p* < 0.0001) were associated with lower changes in VO_2_ max in both sexes. **Conclusions**: Our findings suggest that females and individuals with specific comorbidities benefit less from cardiac rehabilitation and support the creation of personalized rehabilitation programs.

## 1. Introduction

Cardiovascular disease remains the leading cause of death [1]. Accordingly, cardiac rehabilitation, including exercise and risk factors modification following myocardial infarction or cardiovascular revascularization, is a class I recommendation according to the American Heart Association [2]. Nevertheless, it remains underutilized, with participation rates ranging from 10% to 50%, despite strong evidence for reducing cardiovascular mortality, myocardial infarction, hospitalizations, and improving quality of life [2,3,4,5]. Key barriers include lack of awareness of its benefits, as well as travel distance, functional limitations, and low perceived need [6,7,8].

Previous work investigated sex-specific differences concerning cardiac rehabilitation [9]. Females were 12% less likely to be referred, enrolled in, or complete cardiac rehabilitation compared to males [9,10,11,12]. Despite lower participation rates, women may experience greater mortality benefits from cardiac rehabilitation [9,13,14]. Outpatient programs generally improve peak aerobic capacity and maximal oxygen consumption (VO_2_ max), which are linked to reduced hospital readmission and mortality [15,16,17,18]. However, females consistently show lower VO_2_ max both before and after rehabilitation, as well as smaller gains in the 6 min walking test [16,19,20], suggesting a need for more tailored programs with increased training frequency [21,22]. Females may also benefit from peer support and psychosocial services, and participation improves when remote options are available [23]. Furthermore, females demonstrate smaller improvements in systolic blood pressure and perceived health compared with males, underscoring the importance of personalized rehabilitation strategies [24].

While sex-specific differences in cardiac rehabilitation outcomes have been reported, prior studies are often limited by heterogeneous programs or insufficient consideration of comorbidities. Given these observations, the primary aim of this study was to investigate whether females and males derive different benefits from cardiac rehabilitation, assessed by estimated VO_2_ max, within a homogeneous outpatient cohort. A secondary aim was to examine the influence of comorbidities on post-rehabilitation estimated VO_2_ max, with the goal of informing more personalized rehabilitation recommendations.

## 2. Materials and Methods

### 2.1. Study Participants and Consent

This retrospective analysis of previously collected clinical data included patients treated at a cardiological rehabilitation center in Bad Schallerbach, Upper Austria, between July 2007 and July 2013. Ethics approval was obtained from the Medical University of Vienna (EK 1894/2013), and all procedures complied with the Declaration of Helsinki. Participants completed a three-week phase II cardiac rehabilitation program following a prior cardiovascular event. Individuals were excluded if they did not adhere to the training plan (no exercise testing or fewer than two sessions), experienced a cardiac event during rehabilitation, had prior rehabilitation, or had missing data.

### 2.2. Obtained Data

Before rehabilitation, all patients underwent a standardized assessment of fitness and overall health, including medical history, current medication, laboratory tests, resting and stress ECG, and echocardiography to determine left ventricular ejection fraction (EF). Clinical check-ups with medical evaluation and optimization of pharmacological therapy, as well as psychological support took place. Psychological counseling was available throughout the program to address emotional well-being, stress management, and adherence to lifestyle changes. EF was analyzed as a continuous variable and categorized as normal (>55%), mildly reduced (45–54%), moderately reduced (30–44%), or severely impaired (<30%) [25]. Laboratory analyses included blood count, liver and kidney function, fasting glucose, HbA1c, inflammatory markers, lipid profile, cardiac enzymes, and B-type natriuretic peptide, performed locally according to Austrian quality standards. Maximal heart rate was estimated as 220 − age (±15%), and resting heart rate was measured after at least 5 min of seated rest.

### 2.3. Exercise Protocol

Before and after the rehabilitation program, participants completed a stationary bicycle ergometer stress test, starting at 25 W and increasing by 25 W every 2 min until maximum intensity or termination criteria were met, followed by a 5 min recovery at 25 W. ECG and blood pressure were monitored throughout. Termination criteria included significant arrhythmia, ECG changes, abnormal blood pressure, technical issues, performance decline, exhaustion, patient request, angina, poor perfusion, CNS symptoms, and dyspnea. Changes in physical performance were assessed by comparing initial and final tests.

Based on baseline performance, specialists customized rehabilitation programs including cardiovascular gymnastics, hiking, bicycle ergometer, or motorized therapy for mobility-restricted patients. Training employed pulse wave-triggered (heart rate-based), intensity-triggered (constant intensity), or interval (40 s low/20 s high intensity) methods. Patients trained with a physiotherapist 10–18 min daily, plus 3–4 supervised 40–60 min sessions per week at 70–85% maximum heart rate, with 10 min warm-up and recovery. Non-supervised individual sessions were also available but were not documented. Training duration was recorded as total minutes.

All patients received individualized dietary counseling from nutritionists during their rehabilitation stay.

### 2.4. Outcome Measurement

In order to measure the success of the training, the variable VO_2_ max was used, which indicates the maximum rate of oxygen uptake and consumption per minute during ergometer training. It is a fundamental parameter in assessing the endurance capacity and thus the physical fitness of a person. We expressed VO_2_ max relative to kilogram bodyweight. However, the VO_2_ max had to be calculated, as no direct measurement was possible, and since there are sex differences in VO_2_ max, we used a sex-adjusted formula according to Uth et al. [26], described as VO_2_ max = body mass × PF × (maximal heart rate/heart rate at rest), where PF denotes the proportionality factor between heart rates at maximum exercise and rest (maximal heart rate/heart rate at rest) and the mass-specific VO_2_ max, and is defined as 14.5 mL·min^−1^·kg^−1^ for females and 15.3 mL·min^−1^·kg^−1^ for males.

### 2.5. Statistics

Binary variables were reported as absolute and relative frequencies. Continuous variables were reported with means and standard deviations. In order to compare differences, binary variables were tested with a x^2^-test. For continuous variables, a Student’s *t*-test was used.

The relationships between the covariates and the main endpoint were checked with univariate baseline-adjusted analysis of variance (for binary variables) or with univariate baseline-adjusted linear regression models (for continuous variables). Beforehand, the Shapiro test was used to check for normality and Levene’s test was used to check for equality of variances. Covariates that proved significant at α = 0.05 were included in a multivariable linear regression model. Variable selection was performed using backward elimination based on the Akaike information criterion (AIC) to balance model fit and parsimony while minimizing overfitting. This approach is commonly used in observational studies with multiple correlated predictors. The models were adjusted for baseline VO_2_ max, comorbidities, and training intensity. Lastly for the significant findings, the analyses were repeated, stratified by sex. Furthermore, Cohen’s *d* was calculated as a standardized measure of effect size to quantify the magnitude of differences between groups, independently of sample size. Effect sizes were interpreted according to conventional thresholds, with values of approximately 0.2 indicating small, 0.5 moderate, and ≥0.8 large effects.

## 3. Results

In Table 1, we report the baseline characteristics of our study cohort (n = 3239 patients, 81.2% males and 18.8% females). In general, the mean age was 63.94 years for males and 67.18 years for females. Further, the mean BMI was significantly different between males and females (27.52 kg/m^2^ vs. 27.39 kg/m^2^, *p* = 0.002). Next, 45.6% of the males and 41.7% of the females had prior myocardial infarction. Males had a lower frequency of normal ejection fraction compared to females (70.2% vs. 76.0%, *p* = 0.010). Further, the rate of moderate and severe reduced ejection fraction was higher in the male study cohort in comparison to their female counterparts (8.9% vs. 5.7%, *p* = 0.022 and 3.4% vs. 1.6%, *p* = 0.042). Moreover, the rate of coronary heart disease was higher in males compared to females (81.7% vs. 69.4%, *p* < 0.001). In the males a higher frequency of coronary artery bypass graft was seen compared to the females (33.6% vs. 24.2%, *p* < 0.001). Next, concerning comorbidities, the male study cohort reported a lower rate of hypercholesterinaemia, but a higher rate of hyperlipoproteinemia compared to the female study cohort (45.5% vs. 51.7%, *p* = 0.014 and 35.2% vs. 28.3%, *p* = 0.004). Lastly the effect of training was compared between the male and female study cohorts. The sex-adjusted VO_2_ max baseline was higher in males compared to females (26.18 mL/min/kg vs. 23.55 mL/min/kg, *p* < 0.001) and a greater change in VO_2_ max was seen in males (13.90% or 3.64 mL/min/kg change from baseline vs. 11.76% or 2.77 mL/min/kg change from baseline, *p* < 0.001). Moreover, worse EF and a higher overall training duration were found in males compared to females (0.448 vs. 0.324, *p* = 0.001 and 279.9 min vs. 217.79 min, *p* < 0.001).

### Outcome

As a next step, we analyzed how each factor affects the sex-adjusted VO_2_ max change from baseline (Table 2). In our sample, patients with coronary heart disease (β coefficients 0.54; CI 95% 0.10, 0.99; *p* = 0.017) and patients with hypercholesterolemia (β coefficients 0.54; CI 95% 0.18, 0.90; *p* = 0.003) benefited significantly more from the therapy and showed a higher mean VO_2_ max change, while patients with prior heart valve surgery (β coefficients −0.71; CI 95% −1.18, −0.24; *p* = 0.003), cardiomyopathy (β coefficients −1.50, CI95% −2.39, −0.61; *p* < 0.001), or diabetes mellitus (β coefficients −1.30; CI 95% −2.08, −0.53; *p* < 0.001) benefited significantly less and showed a lower mean change VO_2_ max. Higher VO_2_ max change was observed for patients with more training lessons (β coefficients 0.01; CI 95% 0.01, 0.01; *p* < 0.001) and patients with a normal ejection fraction (β coefficients −0.48; CI 95% −0.76, −0.19; *p* < 0.001).

Lastly, a multivariable linear model tested the effect of numerous variables on the mean change in VO_2_ max (Table 3). Similar results to the univariable model were found, except that coronary heart disease and prior heart valve surgery were not significant in the multivariable model. However, female sex was associated with a 1.4-point-lower change in sex-adjusted VO_2_ max after adjustment for different diagnoses and different intensities of therapy (β coefficients −1.41; CI 95% −0.41, −0.10; *p* < 0.001). Moreover, those with a lower VO_2_ max at baseline (β coefficients −0.26; CI 95% −0.41, −0.10 *p* < 0.001), higher ejection fraction (β coefficients −0.34; CI 95% −0.60, −0.08, *p* = 0.010), and the presence of a prior heart valve surgery (β coefficients −0.91; CI 95% −1.44, −0.37; *p* < 0.002) or diabetes mellitus (β coefficients −1.21; CI 95% −1.93, −0.49; *p* < 0.001) also showed lower changes in VO_2_ max. Only hypercholesterolemia was related to a higher change in VO_2_ max (β coefficients 0.44; CI 95% 0.09, 0.79; *p* = 0.014). When stratifying the analyses by sex, lower changes in VO_2_ max were associated with the presence of heart valve surgery in both sexes (male: β coefficients −3.90, *p* < 0.001; female: OR −3.92, *p* < 0.001), but with diabetes mellitus only in males (male: β coefficients −1.42, *p* < 0.001; female: OR −1.03, *p* = 0.089).

## 4. Discussion

In summary, we observed that males may benefit more from cardiac rehabilitation compared to females, as measured by estimated VO_2_ max. Moreover, females might be less likely to meet the training schedule. Lastly, VO_2_ max was positively associated with hypercholesterolemia, but negatively with diabetes mellitus and prior heart valve surgery. While several associations reached statistical significance, interpretation was guided by the magnitude and precision of effect estimates. Some statistically significant findings were characterized by small effect sizes and narrow confidence intervals, suggesting limited clinical relevance despite statistical significance. Therefore, greater weight was placed on effect size and clinical interpretability rather than *p*-values alone.

As mentioned before, cardiac rehabilitation is known to increase the cardiorespiratory fitness level of most individuals, resulting in reduced mortality and hospitalization rate [15,16,17,18]. That is why the current guidelines for myocardial infarction or cardiovascular revascularisation from the American Heart Association include cardiac rehabilitation [2]. Nevertheless, various studies have shown that postmenopausal females have a lower improvement in cardiorespiratory fitness level measured by VO_2_ max and 6 min walking test compared to males [16,19,20]. Also, in our study, the findings suggest lower changes in estimated VO_2_ max in females compared to males. However, females might have a greater benefit in all-cause mortality and cardiovascular mortality than males even if they undergo the same amount of leisure-time physical activity [27]. Sex-related differences in exercise capacity are largely explained by physiological factors, including smaller heart size, lower stroke volume and cardiac output, reduced lung volumes and airway size, lower hemoglobin levels, and a lower proportion of lean body mass in women, all of which limit oxygen delivery and ventilation during exercise [28,29,30,31,32,33,34,35,36,37,38,39,40,41,42]. These disadvantages may outweigh potential benefits, such as more efficient exercise-induced vasodilation, resulting in lower VO_2_ max in females overall. In addition to biological factors, females tend to complete fewer and less intense training sessions, possibly due to older age, higher comorbidity burden, greater perceived exertion, and higher rates of depression, which may reduce adherence to rehabilitation programs [9,43]. Personalized strategies—including psychosocial support, sex-tailored exercise prescriptions, home-based or remote rehabilitation, and motivational interventions—may improve participation and outcomes in females [44,45,46,47]. Although female-focused programs yield only modest fitness gains, they consistently improve participation and mental health, aligning with our findings of persistent sex-based disparities in physical fitness outcomes after cardiac rehabilitation [48].

Nevertheless, in our study, females were less likely to have coronary heart disease and hyperlipoproteinemia. Only in hypercholesterinemia were higher numbers reported in females compared to males. All other comorbidities, including diabetes mellitus, cardiomyopathy, and aortic disorder, did not show sex-specific differences.

In our study, individuals with prior heart valve surgery or diabetes mellitus might not benefit from cardiac rehabilitation as intensely as individuals without these comorbidities. It is already known that individuals with diabetes mellitus experience smaller improvements in metabolic parameters, such as abdominal obesity and lipid profiles, than individuals without diabetes mellitus after training [49]. Further, a metanalysis found that individuals with type 2 diabetes mellitus have lower levels of VO_2_ max compared to individuals without diabetes mellitus [50,51]. Moreover, it is known that people with diabetes mellitus are less likely to undergo cardiac rehabilitation even if it is important [51]. Concerning heart valve surgeries, it is known that they benefit from cardiac rehabilitation [52]. Studies report that individuals who undergo exercise training after heart valve surgery report higher VO_2_ max levels compared to individuals who, after heart valve surgery, did not train [52]. Additionally, a lower mortality and better values in the 6 min walking test was seen after cardiac rehabilitation in heart valve surgery patients [53]. Nevertheless, our study suggested that patients with prior heart valve surgery benefit less from cardiac rehabilitation than other patients. Those with a lower ejection fraction might not gain the same improvement in estimated VO_2_ max in our study compared to those with higher ejection fraction values. However, cardiac rehabilitation is important in heart failure as it improves aerobic capacity and quality of life and reduces hospitalization rate [54,55]. These findings highlight the need for more personalized cardiac rehabilitation programs concerning comorbidity status.

As in every study, we have to report some limitations. Firstly, our study cohort consisted mainly of males. This resulted from the chosen rehabilitation center, which is responsible for individuals who work or have worked as public officers, railway workers, or miners, and these jobs are mostly performed by males. However, in future studies, the sex of the participants should be evenly distributed, and more than one rehabilitation center should be included. Additionally, structured psychosocial data were not available, which may have contributed to residual confounding and to a potential underestimation of rehabilitation effects in female participants. The retrospective design prevented clear identification of the primary referral indication for cardiac rehabilitation because overlapping cardiovascular diagnoses were recorded as clinical characteristics rather than mutually exclusive referral reasons. Next the exercise effect was tested after 3 weeks of training, indicating short-term effects, and only information on the supervised training sessions is reported. Nevertheless, it would be interesting to gain information on medium- and long-term effects of cardiac rehabilitation. Further, including unsupervised training sessions would be important, as well as considering the reasons why training sessions were missed, to understand why females did not benefit as much as males from cardiac rehabilitation. Furthermore, information on heart failure symptoms or brain natriuretic peptide levels is missing. Therefore, no statement regarding the presence of heart failure can be made. Moreover, EF was just measured once; therefore, we could not assess the changes in EF due to rehabilitation. Further, we analyzed data from 2007 to 2013 retrospectively, which may not reflect current practices or recent advancements in cardiac rehabilitation, such as home-based training, and may limit the generalizability of observed sex differences [56,57]. Because VO_2_ max was estimated rather than measured directly, a sex-adjusted prediction formula was required. While this approach reflects standard methodology, it introduces a structural distinction between females and males that may amplify observed sex differences in estimated VO_2_ max. Consequently, the reported sex-specific differences likely reflect both true physiological variations and assumptions inherent to the estimation method and should therefore be interpreted with caution. A further limitation is the lack of detailed information on actual training intensity during unsupervised sessions. Although supervised sessions were delivered according to standardized protocols, variation in exercise behavior outside the clinical setting may have contributed to interindividual differences in training response. This potential source of bias could not be controlled for and should be considered when interpreting the results.

## 5. Conclusions

This study is novel in that it integrates the impact of comorbidities when evaluating outcomes of standard cardiac rehabilitation. Within this retrospective cohort, we found that comorbid conditions significantly influenced changes in estimated functional capacity. Additionally, female participants demonstrated a smaller improvement in estimated functional capacity following cardiac rehabilitation compared with male participants. It suggests that females, as well as individuals with diabetes mellitus or prior heart valve surgery, might benefit less from cardiac rehabilitation, which was measured by estimated VO_2_ max, than the rest of our study cohort. Therefore, more personalized training programs for these individuals should be investigated. Clinicians should assess individual patient needs and adjust therapy plans accordingly to ensure targeted, effective care. Future research should include prospective studies with more recent cohorts to better understand current sex differences in cardiovascular rehabilitation and account for evolving clinical practices.

## Figures and Tables

**Table 1:** Baseline characteristics.

Variable	Males (Percent) (n = 2630)	Females (Percent) (n = 609)	*p*-Value (Males vs. Females)
Age (mean)	63.94 ± 10.53	67.18 ± 11.26	0.431
BMI (mean) in kg/m^2^	27.52 ± 3.86	27.39 ± 4.70	0.002
Myocardial infarction	1026 (46%)	212 (42%)	0.119
Normal ejection fraction	1580 (70%)	387 (76%)	0.010
Slightly reduced ejection fraction	360 (16%)	82 (16%)	1.000
Moderate reduced ejection fraction	201 (9%)	29 (6%)	0.022
Severe reduced ejection fraction	77 (3%)	8 (2%)	0.042
Coronary heart disease	1839 (82%)	353 (69%)	<0.001
Coronary artery bypass graft	756 (34%)	123 (24%)	<0.001
Valvular heart disease	455 (20%)	122 (24%)	0.069
Prior heart valve surgery	417 (19%)	103 (20%)	0.407
Hypercholesterinaemia	1025 (46%)	263 (52%)	0.014
Hyperlipidaemia	792 (35%)	144 (28%)	0.004
Cardiomyopathy	146 (7%)	23 (5%)	0.117
Diabetes mellitus	518 (23%)	114 (22%)	0.810
Aortic disorder	120 (5%)	18 (4%)	0.118
VO_2_ sex-adjusted baseline (mL/min/kg) (mean)	26.18 ± 5.81	23.55 ± 5.02	<0.001
EF (mean)	0.448 ± 0.80	0.324 ± 0.66	<0.001
Total training (min) (mean)	279.89 ± 113.33	217.79 ± 87.87	<0.001
Rehabilitation Outcome			
Change VO_2_ sex-adjusted (mL/min/kg) (mean)	3.64 ± 4.89	2.77 ± 4.28	<0.001

This table presents the basic characteristics of the male and female study cohort. For continuous variables, total numbers and percentages are reported. For numerical variables, the mean and standard deviation are reported. The cohorts were compared by chi^2^ test or *t*-test and the *p*-values are reported. EF = ejection fraction, VO_2_ = maximal oxygen consumption.

**Table 2:** Linear regression model.

Variables	β Coefficients	*p*-Value	95% CI	Cohen’s d	β Coefficients VO_2_-Baseline Adjustment
Total training (min)	0.01	<0.001	0.01; 0.01	3.29	−0.21
Myocardial infarction	0.34	0.074	−0.03; 0.70	1.79	−0.18
Coronary heart disease	0.54	0.017	0.10; 0.99	2.39	−0.18
Coronary artery bypass grafting	0.16	0.419	−0.23; 0.56	0.81	−0.17
Heart valve disorder	−0.73	0.002	−1.20; −0.27	−3.09	−0.19
Prior heart valve surgery	−0.71	0.003	−1.18; −0.24	−2.97	−0.19
Hypercholesterinaemia	0.54	0.003	0.18, 0.90	2.97	−0.17
Hyperlipidaemia	−0.33	0.080	−0.70; 0.04	−1.75	−0.17
cardiomyopathy	−1.50	<0.001	−2.39; −0.61	−3.29	−0.18
Diabetes mellitus	−1.30	<0.001	−2.08; −0.53	−3.29	−0.18
Aortic disorder	−0.15	0.711	−0.96, 0.66	−0.37	−0.17
EF (continuous)	−0.48	<0.001	−0.76, −0.19	−3.29	−0.18

Relationship between the covariates and the main endpoint checked with univariate baseline-adjusted analysis of variance (for binary variables) or with univariate baseline-adjusted regression models (for continuous variables). CI indicates confidence intervals. EF = ejection fraction.

**Table 3:** Final multivariable linear model.

Variable	β Coefficients	*p*-Value	95% CI	Cohen’s d
VO_2_ Baseline sex adjusted	−0.26	<0.001	−0.41; −0.10	−3.29
EF (continuous)	−0.34	0.010	−0.60; −0.08	−2.58
Prior heart valve surgery	−0.91	0.001	−1.44; −0.37	−3.29
Hypercholesterinaemia	0.44	0.014	0.09; 0.79	2.46
cardiomyopathy	−0.81	0.056	−1.64; 0.02	−1.91
Diabetes mellitus	−1.21	<0.001	−1.93; −0.49	−3.29
Total training	0.01	<0.001	0.01; 0.01	3.29
sex	−1.41	<0.001	−2.25, −0.57	−3.29

Here the multivariable regression model is reported, which was reduced by backward selection according to the best Akaike information criterion to retrieve the final model. CI indicates confidence intervals. EF = ejection fraction, VO_2_ = maximal oxygen consumption.

## Data Availability

The data underlying this article cannot be shared publicly due to the privacy of individuals who participated in the study. The data will be shared on reasonable request to the corresponding author.

## Abbreviations

The following abbreviations are used in this manuscript:

CI	confidence interval
EF	ejection fraction
VO_2_ max	maximal oxygen consumption

## References

[1] Tsao C.W., Aday A.W., Almarzooq Z.I., Anderson C.A., Arora P., Avery C.L., Baker-Smith C.M., Beaton A.Z., Boehme A.K., Buxton A.E. (2023). Heart Disease and Stroke Statistics-2023 Update: A Report From the American Heart Association. Circulation.

[2] Simon M., Korn K., Cho L., Blackburn G.G., Raymond C. (2018). Cardiac rehabilitation: A class 1 recommendation. Cleve Clin. J. Med..

[3] Clark R.A., Conway A., Poulsen V., Keech W., Tirimacco R., Tideman P. (2015). Alternative models of cardiac rehabilitation: A systematic review. Eur. J. Prev. Cardiol..

[4] Humphrey R., Guazzi M., Niebauer J. (2014). Cardiac rehabilitation in Europe. Prog. Cardiovasc. Dis..

[5] Dibben G.O., Faulkner J., Oldridge N., Rees K., Thompson D.R., Zwisler A.D., Taylor R.S. (2023). Exercise-based cardiac rehabilitation for coronary heart disease: A meta-analysis. Eur. Heart J..

[6] Leung Y.W., Brual J., Macpherson A., Grace S.L. (2010). Geographic issues in cardiac rehabilitation utilization: A narrative review. Health Place.

[7] Chair S.Y., Chan S.W., Thompson D.R., Leung K.P., Ng S.K., Choi K.C. (2013). Long-term effect of motivational interviewing on clinical and psychological outcomes and health-related quality of life in cardiac rehabilitation patients with poor motivation in Hong Kong: A randomized controlled trial. Clin. Rehabil..

[8] Arena R., Williams M., Forman D.E., Cahalin L.P., Coke L., Myers J., Hamm L., Kris-Etherton P., Humphrey R., Bittner V. (2012). Increasing referral and participation rates to outpatient cardiac rehabilitation: The valuable role of healthcare professionals in the inpatient and home health settings: A science advisory from the American Heart Association. Circulation.

[9] Smith J.R., Thomas R.J., Bonikowske A.R., Hammer S.M., Olson T.P. (2022). Sex Differences in Cardiac Rehabilitation Outcomes. Circ. Res..

[10] Suaya J.A., Stason W.B., Ades P.A., Normand S.L., Shepard D.S. (2009). Cardiac rehabilitation and survival in older coronary patients. J. Am. Coll. Cardiol..

[11] Ritchey M.D., Maresh S., McNeely J., Shaffer T., Jackson S.L., Keteyian S.J., Brawner C.A., Whooley M.A., Chang T., Stolp H. (2020). Tracking Cardiac Rehabilitation Participation and Completion Among Medicare Beneficiaries to Inform the Efforts of a National Initiative. Circ. Cardiovasc. Qual. Outcomes.

[12] Li S., Fonarow G.C., Mukamal K., Xu H., Matsouaka R.A., Devore A.D., Bhatt D.L. (2018). Sex and Racial Disparities in Cardiac Rehabilitation Referral at Hospital Discharge and Gaps in Long-Term Mortality. J. Am. Heart Assoc..

[13] Dunlay S.M., Pack Q.R., Thomas R.J., Killian J.M., Roger V.L. (2014). Participation in cardiac rehabilitation, readmissions, and death after acute myocardial infarction. Am. J. Med..

[14] Goel K., Lennon R.J., Tilbury R.T., Squires R.W., Thomas R.J. (2011). Impact of cardiac rehabilitation on mortality and cardiovascular events after percutaneous coronary intervention in the community. Circulation.

[15] Witvrouwen I., Pattyn N., Gevaert A.B., Possemiers N., Van Craenenbroeck A.H., Cornelissen V.A., Beckers P.J., Vanhees L., Van Craenenbroeck E.M. (2019). Predictors of response to exercise training in patients with coronary artery disease-a subanalysis of the SAINTEX-CAD study. Eur. J. Prev. Cardiol..

[16] Rengo J.L., Khadanga S., Savage P.D., Ades P.A. (2020). Response to Exercise Training During Cardiac Rehabilitation Differs by Sex. J. Cardiopulm. Rehabil. Prev..

[17] De Schutter A., Kachur S., Lavie C.J., Menezes A., Shum K.K., Bangalore S., Arena R., Milani R.V. (2018). Cardiac rehabilitation fitness changes and subsequent survival. Eur. Heart J. Qual. Care Clin. Outcomes.

[18] Vanhees L., Fagard R., Thijs L., Amery A. (1995). Prognostic value of training-induced change in peak exercise capacity in patients with myocardial infarcts and patients with coronary bypass surgery. Am. J. Cardiol..

[19] Nguyen C.H., Thomas S.G., Marzolini S. (2021). Factors Associated with Change in Cardiovascular Fitness for Patients with Peripheral and Coronary Artery Disease in Cardiac Rehabilitation. J. Cardiopulm. Rehabil. Prev..

[20] Bianchi S., Maloberti A., Peretti A., Garatti L., Palazzini M., Occhi L., Bassi I., Sioli S., Biolcati M., Giani V. (2021). Determinants of Functional Improvement After Cardiac Rehabilitation in Acute Coronary Syndrome. High. Blood Press. Cardiovasc. Prev..

[21] Savage P.D., Antkowiak M., Ades P.A. (2009). Failure to improve cardiopulmonary fitness in cardiac rehabilitation. J. Cardiopulm. Rehabil. Prev..

[22] Montero D., Lundby C. (2017). Refuting the myth of non-response to exercise training: ‘non-responders’ do respond to higher dose of training. J. Physiol..

[23] Beatty A.L., Beckie T.M., Dodson J., Goldstein C.M., Hughes J.W., Kraus W.E., Martin S.S., Olson T.P., Pack Q.R., Stolp H. (2023). A New Era in Cardiac Rehabilitation Delivery: Research Gaps, Questions, Strategies, and Priorities. Circulation.

[24] Vidal-Almela S., Marçal I.R., Wong J., Terada T., Nguyen B.O., Joensen A.M., Mills M.T., Bittman J., Prud’Homme D., Reed J.L. (2024). Sex Differences in Changes in Cardiorespiratory Fitness and Additional Health Outcomes Following Exercise Training in Adults with Atrial Fibrillation: A Systematic Review and Meta-Analysis. J. Cardiopulm. Rehabil. Prev..

[25] Harkness A., Ring L., Augustine D.X., Oxborough D., Robinson S., Sharma V. (2020). Normal Reference Intervals for Cardiac Dimensions and Function for Use in Echocardiographic Practice: A Guideline from the British Society of Echocardiography. Echo Res. Pract..

[26] Uth N. (2005). Gender difference in the proportionality factor between the mass specific VO_2max_ and the ratio between HR_max_ and HR_rest_. Int. J. Sports Med..

[27] Ji H., Gulati M., Huang T.Y., Kwan A.C., Ouyang D., Ebinger J.E., Casaletto K., Moreau K.L., Skali H., Cheng S. (2024). Sex Differences in Association of Physical Activity with All-Cause and Cardiovascular Mortality. J. Am. Coll. Cardiol..

[28] Al-Mallah M.H., Juraschek S.P., Whelton S., Dardari Z.A., Ehrman J.K., Michos E.D., Blumenthal R.S., Nasir K., Qureshi W.T., Brawner C.A. (2016). Sex Differences in Cardiorespiratory Fitness and All-Cause Mortality: The Henry Ford ExercIse Testing (FIT) Project. Mayo Clin. Proc..

[29] Gulati M., Black H.R., Shaw L.J., Arnsdorf M.F., Merz C.N., Lauer M.S., Marwick T.H., Pandey D.K., Wicklund R.H., Thisted R.A. (2005). The prognostic value of a nomogram for exercise capacity in women. N. Engl. J. Med..

[30] Myers J., Prakash M., Froelicher V., Do D., Partington S., Atwood J.E. (2002). Exercise capacity and mortality among men referred for exercise testing. N. Engl. J. Med..

[31] Dominelli P.B., Molgat-Seon Y. (2022). Sex, gender and the pulmonary physiology of exercise. Eur. Respir. Rev..

[32] Janssen I., Heymsfield S.B., Wang Z.M., Ross R. (2000). Skeletal muscle mass and distribution in 468 men and women aged 18-88 yr. J. Appl. Physiol..

[33] Lang R.M., Badano L.P., Mor-Avi V., Afilalo J., Armstrong A., Ernande L., Flachskampf F.A., Foster E., Goldstein S.A., Kuznetsova T. (2015). Recommendations for cardiac chamber quantification by echocardiography in adults: An update from the American Society of Echocardiography and the European Association of Cardiovascular Imaging. J. Am. Soc. Echocardiogr..

[34] Diaz-Canestro C., Pentz B., Sehgal A., Yang R., Xu A., Montero D. (2022). Lean body mass and the cardiovascular system constitute a female-specific relationship. Sci. Transl. Med..

[35] Beltrame T., Villar R., Hughson R.L. (2017). Sex differences in the oxygen delivery, extraction, and uptake during moderate-walking exercise transition. Appl. Physiol. Nutr. Metab..

[36] Roberts B.M., Nuckols G., Krieger J.W. (2020). Sex Differences in Resistance Training: A Systematic Review and Meta-Analysis. J. Strength. Cond. Res..

[37] Newman A.B., Kupelian V., Visser M., Simonsick E.M., Goodpaster B.H., Kritchevsky S.B., Tylavsky F.A., Rubin S.M., Harris T.B. (2006). Strength, but not muscle mass, is associated with mortality in the health, aging and body composition study cohort. J. Gerontol. A Biol. Sci. Med. Sci..

[38] Santisteban K.J., Lovering A.T., Halliwill J.R., Minson C.T. (2022). Sex Differences in VO_2max_ and the Impact on Endurance-Exercise Performance. Int. J. Environ. Res. Public Health.

[39] Schmidt W., Prommer N. (2010). Impact of alterations in total hemoglobin mass on VO˙_2max_. Exerc. Sport. Sci. Rev..

[40] Murphy W.G. (2014). The sex difference in haemoglobin levels in adults-mechanisms, causes, and consequences. Blood Rev..

[41] Jelkmann W. (2011). Regulation of erythropoietin production. J. Physiol..

[42] Shahani S., Braga-Basaria M., Maggio M., Basaria S. (2009). Androgens and erythropoiesis: Past and present. J. Endocrinol. Investig..

[43] Senoo K., Kaneko H., Ueno K., Suzuki Y., Okada A., Fujiu K., Jo T., Takeda N., Morita H., Kamiya K. (2024). Sex Differences in the Association Between Depression and Incident Cardiovascular Disease. JACC Asia.

[44] Beckie T.M., Beckstead J.W., Schocken D.D., Evans M.E., Fletcher G.F. (2011). The effects of a tailored cardiac rehabilitation program on depressive symptoms in women: A randomized clinical trial. Int. J. Nurs. Stud..

[45] Jolly K., Taylor R., Lip G.Y., Greenfield S., Raftery J., Mant J., Lane D., Jones M., Lee K.W., Stevens A. (2007). The Birmingham Rehabilitation Uptake Maximisation Study (BRUM). Home-based compared with hospital-based cardiac rehabilitation in a multi-ethnic population: Cost-effectiveness and patient adherence. Health Technol. Assess..

[46] Pepera G., Antoniou V., Su J.J., Lin R., Batalik L. (2024). Comprehensive and personalized approach is a critical area for developing remote cardiac rehabilitation programs. World J. Clin. Cases.

[47] McDonagh S.T., Dalal H., Moore S., Clark C.E., Dean S.G., Jolly K., Cowie A., Afzal J., Taylor R.S. (2023). Home-based versus centre-based cardiac rehabilitation. Cochrane Database Syst. Rev..

[48] Osumi A., Kanejima Y., Kitamura M., Ishihara K., Izawa K.P. (2025). Effects of cardiac rehabilitation on female patients with heart disease: A systematic review and meta-analysis. Maturitas.

[49] Laddu D.R., Ozemek C., Hauer T.L., Rouleau C.R., Campbell T.S., Wilton S.B., Aggarwal S., Austford L., Arena R. (2020). Cardiometabolic responses to cardiac rehabilitation in people with and without diabetes. Int. J. Cardiol..

[50] Macedo A.C.P., Schaan C.W., Bock P.M., Pinto M.B., Botton C.E., Umpierre D., Schaan B.D. (2023). Cardiorespiratory fitness in individuals with type 2 diabetes mellitus: A systematic review and meta-analysis. Arch. Endocrinol. Metab..

[51] Gadager B.B., Tang L.H., Ravn M.B., Doherty P., Harrison A., Christensen J., Taylor R.S., Zwisler A.D., Maribo T. (2022). Benefits of cardiac rehabilitation following acute coronary syndrome for patients with and without diabetes: A systematic review and meta-analysis. BMC Cardiovasc. Disord..

[52] Akpinar F.M., Oral A. (2023). Does Exercise-Based Cardiac Rehabilitation Reduce Mortality and Hospitalization Rates After Heart Valve Surgery?: A Cochrane Review Summary with Commentary. Am. J. Phys. Med. Rehabil..

[53] Xue W., Xinlan Z., Xiaoyan Z. (2022). Effectiveness of early cardiac rehabilitation in patients with heart valve surgery: A randomized, controlled trial. J. Int. Med. Res..

[54] Rengo J.L., Savage P.D., Barrett T., Ades P.A. (2018). Cardiac Rehabilitation Participation Rates and Outcomes for Patients with Heart Failure. J. Cardiopulm. Rehabil. Prev..

[55] Long L., Mordi I.R., Bridges C., Sagar V.A., Davies E.J., Coats A.J., Dalal H., Rees K., Singh S.J., Taylor R.S. (2019). Exercise-based cardiac rehabilitation for adults with heart failure. Cochrane Database Syst. Rev..

[56] Thomas R.J. (2024). Cardiac Rehabilitation—Challenges, Advances, and the Road Ahead. N. Engl. J. Med..

[57] Lawton J.S., Tamis-Holland J.E., Bangalore S., Bates E.R., Beckie T.M., Bischoff J.M., Bittl J.A., Cohen M.G., DiMaio J.M., Don C.W. (2022). 2021 ACC/AHA/SCAI Guideline for Coronary Artery Revascularization: A Report of the American College of Cardiology/American Heart Association Joint Committee on Clinical Practice Guidelines. J. Am. Coll. Cardiol..

